# Wormian bones: expanded differential diagnosis and implications for abnormal head shape in infancy

**DOI:** 10.1007/s00381-025-06978-z

**Published:** 2026-01-03

**Authors:** Noah E. Alter, James L. Rogers, Marcelina Puc, Anthony Hoang, Izabela Galdyn, Christopher M. Bonfield, Matthew Pontell, Michael Golinko

**Affiliations:** 1 Department of Plastic Surgery, Vanderbilt University Medical Center, Nashville, TN USA; 2 Vanderbilt University School of Medicine, Nashville, TN USA; 3 Thomas F. Frist, Jr. College of Medicine at Belmont University, Nashville, TN USA; 4 Vanderbilt University, Nashville, TN USA; 5 Division of Pediatric Plastic Surgery, Monroe Carell Jr. Children’s Hospital, Nashville, TN USA; 6 Department of Neurosurgery, Vanderbilt University Medical Center, Nashville, TN USA

**Keywords:** Wormian bones, Cranial morphology, Abnormal Head Shape, Brachycephaly, Craniosynostosis

## Abstract

**Purpose:**

Wormian bones (WB) are accessory ossicles that develop within cranial sutures. While typically benign, their presence in large numbers has been associated with various genetic and developmental disorders. This study aims to characterize the prevalence, anatomical distribution, and clinical associations of WB in a pediatric population undergoing cranial CT imaging.

**Methods:**

A retrospective review was conducted at Monroe Carell Jr. Children’s Hospital at Vanderbilt. Pediatric patients aged 0 to 18 years who underwent cranial CT imaging for any clinical indication were included. WB were radiologically confirmed, and demographic data, cephalic index, and comorbidities were collected and analyzed.

**Results:**

Among the 13,519 patients who underwent cranial CT imaging, 77 (0.57%) had radiologically confirmed WB, totaling 476 ossicles. The prevalence increased to 2.1% when examining our clinic cohort over a 5-year period. The lambdoid suture was the most common site (343/476, 72.1%), followed by the posterior fontanelle (53/476, 11.1%). Multiple WB (≥ 2) represented the most common phenotype (52/77, 67.5% of cases), and 13 patients (16.8%) had at least one associated skeletal or craniofacial condition, most commonly craniosynostosis (10/13, 76.9%). Cephalic index analysis demonstrated a predominance of brachycephaly (54/77, 70.1%).

**Conclusion:**

This study presents a comprehensive evaluation of WB in a large pediatric cohort. WB may co-exist with craniosynostosis or other skeletal conditions such as osteogenesis imperfecta. Given the greater prevalence of WB compared to craniosynostosis (2.1%: clinic cohort & 0.57%: institutional cohort vs. 0.05%), clinicians should consider WB in the differential diagnosis of abnormal head shape in infancy, particularly in a subspecialist practice.

## Introduction

Wormian bones (WB) or intrasutural bones are accessory ossicles that develop within the cranial sutures, most commonly along the lambdoid suture [1]. These bones are considered benign anatomical variants and are not associated with neurocognitive impairment [2]. While often incidentally discovered, WB, particularly in large numbers, have been associated with various genetic and developmental disorders including craniosynostosis, osteogenesis imperfecta, and cleidocranial dysplasia [1–3]. Their frequency and anatomical distribution vary widely across populations, suggesting a combined genetic and environmental influence on their development [4, 5]. Additionally, WB have been noted to influence cranial morphology, raising important diagnostic considerations when evaluating infants with atypical head shape [6]. As such, defining their baseline incidence and anatomical distribution is necessary for distinguishing benign anatomical variants from underlying pathology.

Despite these recognized associations, the epidemiology of WB remains incompletely characterized [7–9]. The existing literature predominantly focuses on infants with syndromic diagnoses, small case series, and animal models, limiting their generalizability [7–10]. Additionally, inconsistencies in reported prevalence, ranging from 9 to 95 percent, may stem from methodological variations in imaging modalities, study design, and/or population demographics [3, 10, 11] Factors such as age, ethnicity, sex, and genetic predisposition have all been proposed as potential influences on WB formation, yet no consensus exist regarding their relative contributions [1, 3, 7–10]. Furthermore, the biological mechanisms underlying WB formation remain elusive, with hypotheses ranging from intrinsic genetic determinants to extrinsic biomechanical forces shaping cranial suture development [1, 2, 4, 6–11].

Given the reliance on neuroimaging to evaluate atypical head shape in infancy, a more comprehensive understanding of WB prevalence, sutural distribution, and demographic correlates is warranted. This study aims to characterize the prevalence, anatomical distribution, and associated demographic features of WB in a large pediatric cohort undergoing cranial CT imaging over a ten-year period, as well as within a focused subspecialty craniofacial clinic cohort.

## Methods

### Study design

Following Institutional Review Board approval (IRB #242162), a retrospective chart review was conducted at Monroe Carell Jr. Children’s Hospital at Vanderbilt. This review included all patients who underwent cranial CT imaging (CPT codes 70450, 70460, and 70470) between January 1, 2014, and December 31, 2024. Patients were identified using Vanderbilt University’s Research Derivative, a clinical repository of electronic medical record (EMR) data. Within this cohort, all patients with documented WB were flagged for further review. Only cases with radiographically confirmed WB were included.

### Inclusion and exclusion criteria

All patients aged 0 to 18 years with radiologically confirmed WB on cranial CT imaging were included. Patients were excluded if WB were mentioned in EMR without imaging confirmation.

### Chart review

A detailed chart review was performed by two independent reviewers (N.A. and J.R.) to confirm the presence and anatomical distribution of WB. Patients were categorized based on the total number of WB, their sutural location, and the primary indication for cranial CT imaging. Demographic variables, including age, sex, and race, were also recorded.

### Measurement of cephalic index

To assess cranial morphology, the cephalic index (CI) was calculated for all patients with CT-confirmed WB. Maximum cranial length was measured as the greatest anteroposterior distance between the glabella and the opisthocranion in the sagittal plane. Maximum cranial width was measured as the greatest transverse distance between the two euryon points in the coronal plane. CI was calculated using the formula: Cephalic Index = (Maximum Cranial Width/Maximum Cranial Length) × 100.

### Imaging and measurement protocol

To ensure consistency, all measurements were conducted using SECTRA PACS radiology software (Sectra AB, Linköping, Sweden). Each measurement was independently performed by two authors (N.A. and J.R.), and the results were averaged to minimize interobserver variability.

### Classification of cranial shapes

Patients were classified into three cranial shape categories based on their cephalic index. CI classification followed the system described by Standring and was aligned with the standards of the Frankfurt Craniometric Conference [12, 13]. A CI less than 75.0 was classified as dolichocephalic, indicating an elongated skull shape. A CI between 75.0 and 80.0 was defined as mesocephalic, representing a medium or proportionally balanced skull. A CI greater than 80.0 was classified as brachycephalic, consistent with a shorter and wider cranial shape. These classifications were used to examine potential relationships between cranial shape and WB presence.

### Clinic-based cohort analysis

To assess the relevance of our findings and translation to a typical craniofacial practice, we conducted a secondary analysis of patients aged 0 to 2 years who presented for the evaluation of abnormal head shape to Monroe Carell Jr. Children’s Hospital Plastic Surgery or Pediatric Neurosurgery clinics between January 1, 2019, and January 1, 2025. Inclusion criteria were based on visit diagnosis codes consistent with cranial shape anomalies, including congenital deformity of the skull or face (Q75.9), abnormal diagnostic imaging of the skull (R93.0), craniosynostosis (Q75.0), plagiocephaly (Q67.4), and other congenital malformations/deformities of skull, face, and jaw (Q67.1, Q67.3, Q67.8, Q75.8). We recorded whether imaging was performed, the indication for imaging, and whether the patient received a diagnosis of WB, craniosynostosis, or another craniofacial anomaly.

### Statistical analysis

All statistical analyses were conducted using IBM SPSS for Windows (Version 20.0, Armonk, NY). Descriptive statistics were used to summarize patient demographics, WB prevalence, anatomical distribution, and cephalic index classifications. Categorical variables, including WB presence by suture location and cranial shape classification, were presented as frequencies and percentages. Continuous variables were reported as means and standard deviations.

## Results

13,519 pediatric patients, aged 0–18, underwent cranial CT imaging between 2014 to 2024. Of these 77 (0.57%) had radiologically confirmed WB, totaling 476 ossicles. The lambdoid suture was the most common site (343/476, 72.1%), followed by the posterior fontanelle (53/476, 11.1%). WB were more frequently observed in males (67.5%), Caucasian patients (54.5%), and infants less than 1 year old (79.2%). Multiple WB (≥ 2) represented the most common phenotype, observed in 67.5% of cases.

**Fig. 1:**
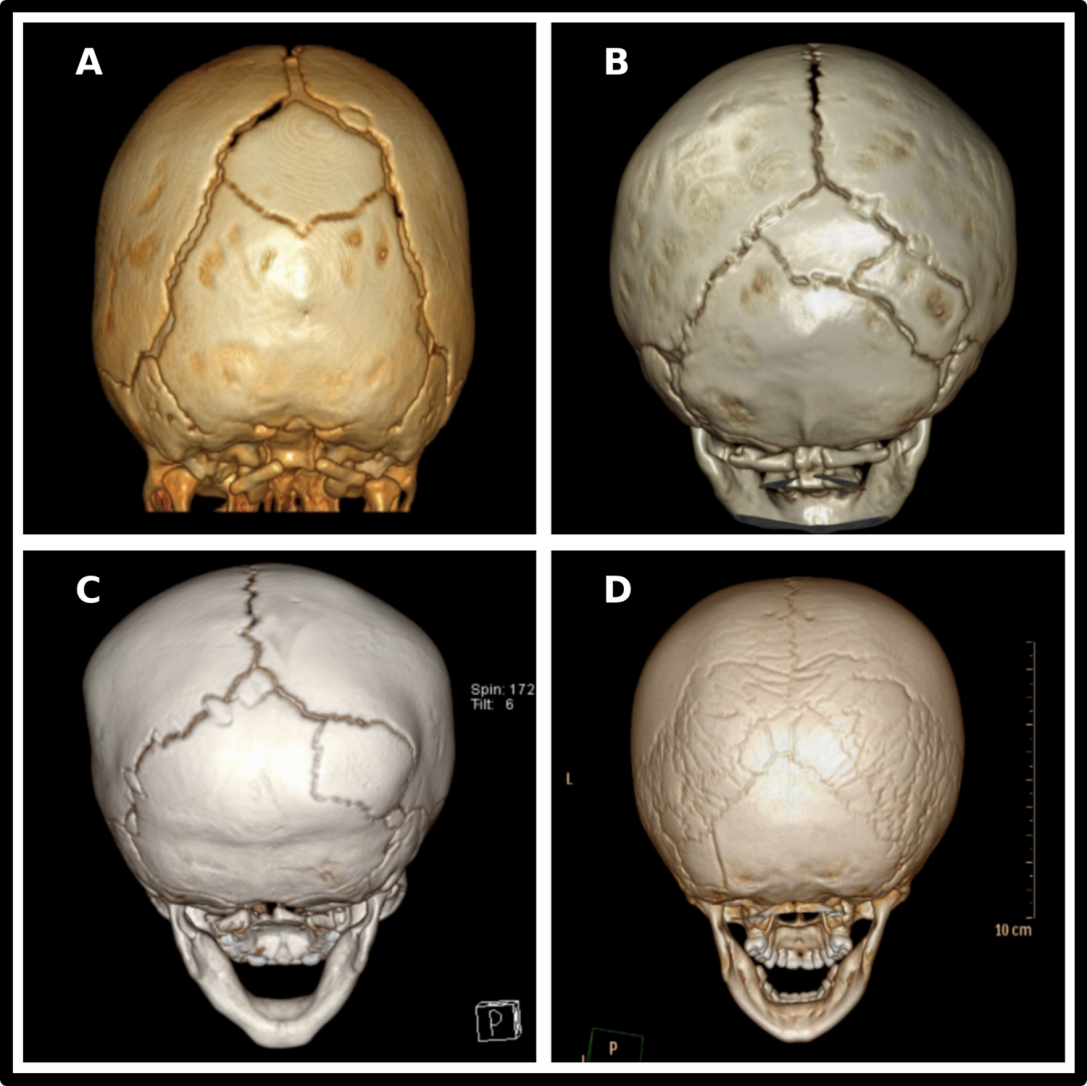
3D-Reconstructed CT Images Demonstrating the Spectrum of Wormian Bone Patients. (**a**) Patient with a single wormian bone located in the posterior fontanelle. (**b**) Patient with two wormian bones (posterior fontanelle and right lambdoid suture), representing the median number identified in this study. (**c**) Patient with six wormian bones distributed across the right and left lambdoid sutures and posterior fontanelle, representing the average number identified in this study. (**d**) Patient with seventy-one wormian bones involving the right and left lambdoid sutures and occipital bone; the highest count recorded in this study

Demographic information for the cohort is provided in Table 1. CT images illustrating the spectrum of WB burden are depicted in Fig. 1.

**Table 1:** Institutional cohort demographics (2014–2024)

Patient Demographics (2014–2024)		
**Category**	**Subcategory**	**N (%)**
**Prevalence**	CT Confirmed Wormian Bones	77/13519 (0.6)
**Age (Years)**	< 1	61/77 (79.2)
	1–2	9/77 (11.7)
	2—5	4/77 (5.2)
	5—18	3/77 (3.9)
**Gender**	Male	52/77 (67.5)
	Female	25/77 (32.5)
**Race**	Caucasian	42/77 (54.6)
	African American	6/77 (7.8)
	Asian	1/77 (1.3)
	Hispanic	9/77 (11.7)
	Other	0/77 (0.0)
	Unknown	19/77 (24.7)
**Classification**	Presence of Craniofacial or Skeletal Comorbidity	13/77 (16.8)
**Wormian Bone Data**	Total Wormian Bone Count	476
	Wormain Bones Per Patient (Average)	6.2
	Wormain Bones Per Patient (Median)	2.0

### Age at identification of wormian bones

**Fig. 2:**
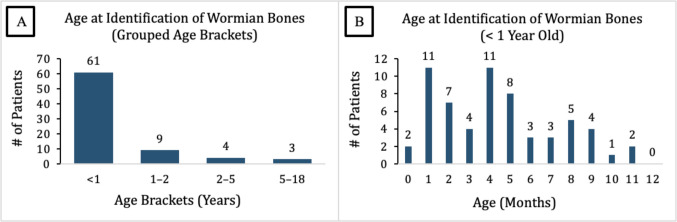
Age at Identification of Wormian Bones. (**a**) Age at identification categorized into grouped age brackets. (**b**) Monthly breakdown of patients under one year of age

WB were most frequently identified during the first year of life, accounting for 61 of 77 cases (79.2%) **[**Fig. 2**]**. The highest detection rates occurred in the 1st and 4th months, with 11 cases each. Beyond 12 months, only 16 cases (20.8%) were identified, with a marked decline in frequency as patient age increased.

### Location of wormian bones

WB were predominantly located in the posterior cranial region, (403/476 bones, 84.7%) **[**Table 2**]**. The lambdoid suture served as the primary site (343 bones, 72.1%), followed by the posterior fontanelle (53 bones, 11.1%).

**Table 2:** Frequency of wormian bones by location

Location		# of Wormian bones
Anterior	Anterior fontanelle	10
	Frontal bone	1
	Total	11
Posterior	Lambdoid suture	343
	Posterior fontanelle	53
	Occipital bone	7
	Total	403
Lateral	Squamous	15
	Mastoid	6
	Sphenoid	2
	Total	23
Vertex	Sagittal suture	31
	Parietal bone	6
	Coronal Suture	2
	Total	39

Together, these two sites comprised 82.6% of all WB identified. In contrast, the lateral and anterior cranial regions were minimally involved, contributing only 4.8% and 2.3% respectively.

### Associated conditions

**Fig. 3:**
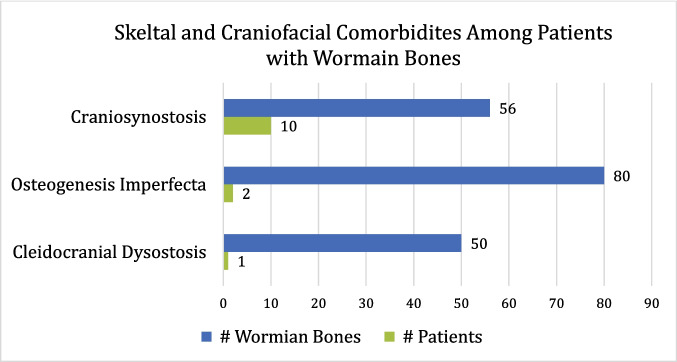
Coexistence of skeletal and craniofacial comorbidities in pediatric patients with wormian bones

13 patients (16.8%) presented with one or more skeletal or craniofacial comorbidity **[**Fig. 3**]**. Craniosynostosis (CS) was the most frequently observed condition, affecting 10 patients and collectively accruing 56 bones. Osteogenesis imperfecta (OI), identified in 2 patients, was associated with the highest WB burden (80 ossicles).

Among the 10 patients with CS, sagittal synostosis was the most common subtype (5/10). The mean cephalic index in this subgroup was 84.4 ± 13.8, with brachycephaly present in 50% (5/10) of patients.

### Cephalic index (CI) classifications

The average CI was 84.3 ± 8.2, with 70.1% (54/77) of patients classified as brachycephalic **[**Table 3**]**. This was followed by mesocephalic individuals (13 patients, 16.9%).

**Table 3:** Cranial shape classifications and measurements

Classification Counts	
**Classification**	**N (%)**
Brachycephalic	54 (70.1)
Mesocephalic	13 (16.9)
Dolichocephalic	10 (13.0)
**Measurement**	**Mean ± SD**
CT Length (mm)	143.1 ± 17.1
CT Width (mm)	120.1 ± 14.4
Cranial Index	84.3 ± 8.2

### CT indications and incidental identification of wormian bones

The most common indication for cranial CT imaging was the evaluation of abnormal head shape (37/77, 48.1%). This was followed by trauma (22/77, 28.6%) and altered mental status (6/77, 7.8%). No CT scans were obtained for the purpose of evaluating WB (0/77, 0.0%). In all cases, WB were identified incidentally.

### Parallel clinic-based cohort analysis

Over a five-year period (2019–2025), 486 patients aged 0 to 2 years presented to our academic craniofacial clinic for the evaluation of abnormal head shape **[**Table 4**]**. Of these, 170 patients (35.0%) underwent cranial CT imaging. 52 patients (10.7%) were diagnosed with CS and 10 (2.1%) had WB. One patient (0.2%) was found to have both CS and WB.

**Table 4:** Frequency of craniosynostosis and wormian bones in a clinic-based cohort presenting with head shape concerns

Imaging Performed	Category	N (%)
	Frequency of Cranial CT imaging	170/486 (35.0)
	Average age at imaging (years)	0.50 ± 0.46
**Indications for Cranial CT Imaging**	**Category**	**N (%)**
	Abnormal head shape	48/170 (28.2)
	Altered mental status	27/170 (15.9)
	Craniosynostosis	26/170 (15.3)
	Hydrocephalus/ventriculomegaly	19/170 (11.2)
	Other	18/170 (10.6)
	Intracranial/extracranial hemorrhage	11/170 (6.5)
	Trauma	8/170 (4.7)
	Genetic	6/170 (3.5)
	VP-shunt placement/dysfunction	5/170 (2.9)
	Tumor	2/170 (1.2)
**Presence of Craniosynostosis**	**Location**	**N (%)**
	Sagittal	21/486 (4.3)
	Coronal	12/486 (2.5)
	Metopic	11/486 (2.3)
	Lambdoid	6/486 (1.2)
	Squamosal	1/486 (0.2)
	Occipomastoid	1/486 (0.2)
	**Total:**	**52/486 (10.7)**
**Presence of Wormian bones**	**Location**	**N (%)**
	Lambdoid suture	6/486 (1.2)
	Sagittal suture	2/486 (0.4)
	Anterior fontanelle	1/486 (0.2)
	Posterior fontanelle	1/486 (0.2)
	**Total:**	**10/486 (2.1)**

## Discussion

Wormian bones (WB) are accessory ossicles that develop within the cranial sutures, most commonly along the lambdoid suture [1]. These bones were first systematically described by the Danish anatomist Olaus Worm in the seventeenth century and subsequently named in his honor [1, 14]. Historically regarded as benign anatomical variants, their presence in large numbers has been associated with a range of genetic, metabolic, and developmental conditions, including craniosynostosis (CS), osteogenesis imperfecta (OI), and cleidocranial dysplasia (CD) [1–3, 15–17].

While the etiology of WB remains incompletely understood, a multifactorial origin involving both genetic predisposition and biomechanical influences such as abnormal dural tension and altered suture dynamics is supported [4, 5, 16–18]. Isolated WB are frequently encountered in pediatric neuroimaging and are often incidental; however, an elevated WB burden may serve as a phenotypic marker of broader skeletal or genetic disease [4, 5, 10, 14–18]. In such cases, WB may serve as an implicit indicator of underlying metabolic, developmental, or genetic conditions and should be considered in the broader diagnostic workup of atypical head shape.

This study aimed to characterize the prevalence, anatomical distribution, and demographic features of WB in a large pediatric cohort undergoing cranial CT imaging at a single institution. Additionally, we examined the frequency of WB in a clinic-based cohort of infants presenting with abnormal head shape. Among the 13,519 pediatric patients who underwent cranial CT imaging between 2014 to 2024, 77 (0.57%) had radiologically confirmed WB, totaling 476 ossicles. The lambdoid suture was the most frequent site (72.1%), followed by the posterior fontanelle (11.1%). Affected individuals were predominantly Caucasian (54.5%), male (67.5%), and younger than 12 months of age (79.2%). 13 patients (16.8%) had at least one associated skeletal or craniofacial condition, most commonly CS (76.9%). Multiple WB (≥ 2) were present in 67.5% of cases, and brachycephaly was the most prevalent cranial morphology (70.1%). This finding was unexpected, as the senior author had anecdotally observed an association between dolichocephalic head shape and occipital WB in the absence of craniosynostosis. A possible explanation may involve clinician bias and imaging thresholds: given that the primary clinical concern is often to rule out craniosynostosis, providers may be more likely to obtain imaging in patients with brachycephalic morphology. In our craniofacial clinic cohort of 486 infants evaluated for abnormal head shape, 52 (10.7%) were diagnosed with CS and 10 (2.1%) were found to have WB.

The observed prevalence of WB in our institutional cohort (0.57%) was markedly lower than rates reported in osteological studies, which range from 9 to 95% across global populations [4, 6, 8, 11, 19]. This discrepancy likely reflects differences in methodology and patient sampling. Prior investigations often relied on dry skull examinations, which are highly sensitive to subtle ossicles but typically involve small, highly selected cohorts [2, 4, 7, 20]. In contrast, our study analyzed a larger and more diverse clinical population. Notably, WB prevalence in our clinic-based cohort (2.1%) was nearly four times higher than our general cohort (0.57%), suggesting that WB are more frequently identified in targeted evaluations than in incidental imaging.

WB have demonstrated an increased frequency in certain ethnic groups, most notably in Greek and Chinese populations, suggesting a possible genetic or epigenetic basis for their development [4, 6, 20]. Our cohort was composed primarily of Caucasian males, reflecting the demographic makeup of our institution’s catchment area. While this homogeneity strengthens internal consistency, it inherently limits external generalizability. Movsesian et al. analyzed over 2,000 skulls from 33 ethnic populations and found that the distribution of sutural bones roughly followed patterns of shared ancestry [8]. Groups with similar genetic backgrounds, especially in Southeast Asia, tended to show similar WB patterns, suggesting that genetics may play a role in WB formation. However, others have noted that infant positioning practices may also affect WB development, particularly in cultures where supine sleeping is common [17]. In this context, our observed prevalence (0.57%) of WB may reflect local demographic factors rather than true biological rarity. Future investigations should incorporate multi-ethnic cohorts to disentangle biological variation from sampling biases.

A total of 476 WB were identified in 77 pediatric patients, with a mean of 6.2 ± 11.1 WB per patient. The posterior cranial region, particularly the lambdoid sutures, was the primary location (72.1%). This predilection may be a consequence of dural strain from infants laying on their backs, high mechanical stress, or the lambdoid suture’s role in accommodating occipital growth, all of which could contribute to irregular ossification patterns [1, 6, 10, 17, 20]. Our findings mirror prior pediatric and adult studies, which consistently report high WB concentrations in the lambdoid region, with more sparse distribution across the sagittal, parietomastoid, and coronal sutures [1, 3–6, 10, 11, 14].

While the presence of a single WB typically holds little clinical significance, a high WB burden across multiple sutures has been associated with genetic conditions such as CS, OI, and CD [1–3, 15–17]. In our cohort, 32% of patients presented with a single WB, 39% had between two and five, and 29% had more than five, with individual WB counts ranging from 1 to 71. Greater than 5 WB were most frequently observed in patients with coexisting CS, OI, and CD, further supporting the association between an elevated WB burden and underlying skeletal pathology. A high WB burden may therefore reflect broader metabolic or genetic disturbances in cranial bone formation or suture homeostasis [15–17]. These finding underscore the need for heightened clinician awareness when multiple WB are present.

In our cohort, 79.2% of patients with WB were identified within the first year of life, with detection rates declining sharply thereafter. This pattern echoes the findings of Marti et al. and Pryles et al., who observed greater WB prevalence in younger children and posited that their frequency decreases as cranial sutures fuse or ossify [6, 21]. Similarly, El-Najjar et al. and O’Loughlin et al. reported higher incidences of WB in infants subjected to intentional cranial deformation, suggesting that early mechanical forces may influence both the formation and potential resolution of these ossicles [18, 22]. Whether WB truly regress with age or simply become radiographically indistinct remains unclear. Given that most cranial CT imaging occurs during infancy, it is difficult to determine whether the higher detection rates reflect genuine biological susceptibility or simply increased imaging exposure. Nevertheless, the predominance of early‑life presentation indicates that WB are likely a transient developmental feature, and their presence, especially when numerous, should prompt evaluation for underlying skeletal or suture‑related pathology.

Our cephalic index (CI) analysis revealed that 70.1% of patients with WB exhibited either brachycephalic (CI > 80.0) or severe brachycephalic morphology. Only 16.9% of patients were mesocephalic (CI: 75.0–80.0) and 13.0% dolichocephalic (CI < 75.0). It is important to note that elevated CI values may result from prolonged supine positioning in infancy, a common etiology of deformational brachycephaly, rather than from the presence of WB themselves [17, 23–28]. As prior studies have reported, higher rates of WB are found in infants with conditions that predispose to prolonged recumbency, such as hypotonia and Down syndrome, raising the possibility that WB and brachycephaly may emerge from parallel biomechanical and/or developmental influences [8, 17, 28–31] However, it remains unclear whether WB contribute to altered cranial shape, result from it, or simply coexist within a common mechanical environment. Nevertheless, these findings suggest that WB can occur across the full spectrum of cranial morphologies and are not exclusively associated with CS.

Abnormal head shape remains one of the most common indications for pediatric cranial imaging, particularly in settings where ultra-low dose CT is available. These evaluations are frequently performed to rule out CS [23, 32]. In our study, WB were never the primary indication for cranial CT imaging and were identified nearly four times more often in the craniofacial clinic population than in the institutional cohort. Despite this increased prevalence, we did not find sufficient evidence to support routine CT screening for WB, as no consistent association with previously undiagnosed pathology was identified. Nonetheless, WB should be included in the differential diagnosis when evaluating infants with atypical head shape. Their recognition can help guide appropriate clinical reassurance and may contribute to broader diagnostic considerations in the context of craniofacial anomalies.

This study has several limitations. First, its retrospective design introduces potential selection and documentation biases as WB were never the primary indication for cranial CT imaging. Second, our study population was derived from a single tertiary care center and was predominantly composed of Caucasian males, which limits the generalizability of our findings. Third, although CI and comorbid diagnoses were assessed, we were unable to evaluate the longitudinal progression of WB burden due to the lack of serial imaging. Fourth, WB dimensions were not measured, therefore restricting our ability to assess potential contributions to cranial morphology. Finally, as an observational study, the causality between WB burden and specific diagnoses could not be established.

Despite these limitations, this study represents one of the largest CT-based evaluations of WB to date, offering valuable insights into their prevalence, anatomical distribution, and clinical associations. A major strength lies in the inclusion of both a broad institutional imaging cohort and a targeted craniofacial clinic population, allowing for direct comparison between incidental and referral-based detection.

## Conclusion

Wormian bones were identified in 0.57% (77/13,519) of pediatric patients, with a strong predilection for the lambdoid suture (72.1%) and posterior fontanelle (11.1%). Affected individuals were predominantly Caucasian (54.5%), male (67.5%), and under 12 months of age (79.2%). Multiple WB (≥ 2) represented the most common phenotype (67.5%), with WB burden ranging up to 71 ossicles. Within our craniofacial clinic cohort, WB were present in 2.1% (10/486) of infants, nearly four times the rate observed in the general imaging population. Clinicians should recognize WB as a frequently incidental yet potentially informative radiologic finding that should be included in the differential diagnosis of abnormal head shape. Future research should incorporate genetic analyses and longitudinal imaging to clarify the developmental relevance and diagnostic utility of WB.

## Data Availability

No datasets were generated or analysed during the current study.
